# P194: Preoperative screening of patients – a small step for the hospital, a big step for epidemiology

**DOI:** 10.1186/2047-2994-2-S1-P194

**Published:** 2013-06-20

**Authors:** A Moldovan, L Dobrin, I Cotoara, B Angheloiu, G Proca, B Moldovan

**Affiliations:** 1 SPCIN, "Sf.Constantin" Hospital, Brasov, Romania; 2 Laboratory, "Regina Maria" Campus, Brasov, Romania; 3 Surgery , "Sf.Constantin" Hospital, Brasov, Romania

## Introduction

Preoperative screening, associated with adequate isolation measures of patients colonized with multidrug-resistant germs and implementation of effective hand hygiene measures – according to the model HUG Geneva- is a major goal of the activity in Hospital “Sf. Constantin”, ensuring effective epidemiological control, adaptation of preoperative antibiotic prophylaxis, optimization of the medical act and, as final result, decreased risk of morbidity / mortality.

## Objectives

Establishing the actual incidence of colonization with methicillin-resistant *Staphylococcus aureus* – MRSA and gram-negative bacteria producing Extended-Spectrum Beta-LactamasesESBL in a sample of population represented by hospitalized patients and monitoring the correct implementation of epidemiological isolation measures and hygiene of hospital staff and environment.

## Methods

Observational and statistical study

## Results

from 01.01.2012 to 31.12.2012 MRSA and ESBL screening was conducted on a number of 2145 admitted patients (sample collection is done before hospitalization, with results available at the time of admission).The detected incidence of MRSA colonization is 10.29% and ESBL 7.8%, with no statistically significant statistic differences between the various surgical or oncology specialties.The screening results overlapped the pre-or intraoperative bacteriological examinations in 27 % of cases. The existence of risk factors for colonization was detected in 54 % of cases.Knowing particular colonization allows initiation of appropriate containment measures in 94 % of cases and administration of adequate preoperative antibiotic prophylaxis to a percentage of 78% of patients operated.Self-control samples collected from the hospital personnel and hospital environment, monthly, have not revealed nosocomial germs.The nosocomial infection rate detected was 0.07%.

## Conclusion

Along with the other measures implemented in the hospital on good medical practice and hand hygiene, preoperative screening is one of the chain links preventing occurrence of nosocomial infections.

## Disclosure of interest

None declared

